# Trends in transfusion of trauma victims - evaluation of changes in clinical practice

**DOI:** 10.1186/1757-7241-19-23

**Published:** 2011-04-11

**Authors:** Anders R Nakstad, Nils O Skaga, Johan Pillgram-Larsen, Berit Gran, Hans E Heier

**Affiliations:** 1 Department of Anaesthesia, Oslo University Hospital, Oslo, Norway; 2 Air Ambulance Department, Oslo University Hospital, Oslo, Norway; 3 Department of Cardiothoracic Surgery, Oslo University Hospital, Oslo, Norway; 4 Blood Bank of Oslo, Department of Immunology and Transfusion Medicine, Oslo University Hospital, Oslo, Norway; 5 University of Oslo, Faculty of Medicine, Oslo, Norway

## Abstract

**Background:**

The present study was performed to compare blood product consumption and clinical results in consecutive, unselected trauma patients during the first 6 months of year 2002, 2004 and 2007.

**Methods:**

Clinical data, blood product consumption, lowest haemoglobin values on day 1-10 after admission, and 30-day mortality were extracted from in-hospital trauma registry and the blood bank data base. The subpopulation of massively transfused patients was identified and analysed separately.

**Results:**

The total number of admitted trauma patients increased by 48% from 2002 to 2007, but the clinical data remained essentially unchanged. The mean number of erythrocyte units given day 1-10 decreased insignificantly from 9.4 in 2002 to 6.8 in 2007. New Injury Severity Score (NISS) increased in transfused and massively transfused patients, but not significantly. The number of patients transfused with plasma increased and the mean ratio of erythrocyte to plasma units transfused decreased by about 50%. The mean haemoglobin value in transfused patients on day 2 after admittance was significantly lower in 2007 than in 2002, while that on day 10 was significantly higher in 2007 than in 2002 and 2004. There was no change of 30-day survival from 2002 to 2007.

**Conclusions:**

Significant changes of transfusion practice occurred during the past decade, probably as a result of increased focus on haemostasis and more precise criteria for transfusion. Despite a lower consumption of erythrocytes in 2007 than in 2002 and 2004, the mean haemoglobin level of transfused patients was higher on day 10 in 2007. The low number of transfused patients in this material makes evaluation of effect on survival difficult. Larger studies with strict control of all influencing factors are needed.

## Background

Intravenous volume replacement and transfusion policies in bleeding trauma patients have traditionally been based largely on local tradition and current opinions [1]. The main focus was on restoring intravascular volume and heamatocrit, thereby securing oxygen transport capacity. During recent years several studies have suggested that early and aggressive use of prohaemostatic blood components (thrombocyte concentrates, fresh frozen plasma) may improve the survival rate significantly [2-6]. However, studies also have been published which fail to support this view [7-9]. The significance of aggressive prohaemostatic transfusion regimens remains unsettled, and there is a need for further studies to extend current knowledge [10]. Parallel to the evolving knowledge in transfusion therapy surgical and angio-embolization techniques improve and patient groups may change. In this study we wanted to describe the change in trauma transfusion practice at Oslo University Hospital - Ullevål (OUHU) during a 5-year period and to evaluate if there is any visible clinical effect of anticipated changes in transfusion practice. Because blood products are a limited resource, we also wanted to evaluate how the total consumption of blood products in trauma care has changed.

## Methods

### Population and study database

The OUHU is the trauma referral centre in a mixed urban and rural area with 2.5 million inhabitants and the major trauma hospital for 550 000 citizens in the Norwegian capital Oslo. Approximately 40% of admitted trauma patients have Injury Severity Score (ISS) >15 [11]. The volume-criterion for a Level-1 trauma hospital of 500 patients in this group per year is met [12]. The OUHU blood bank facility serves all hospitals in the community of Oslo and provides about 25% of the total consumption of blood products in Norway. To gain a representative five-year period all trauma patients admitted during the first six months of the years 2002, 2004 and 2007 were included. These periods were chosen because they represent stable periods between possible local changes in transfusion practice. Clinical data from the first 10 days of treatment and 30-day mortality data were sampled. Length of stay in the hospital (LOS) and LOS in the intensive care unit (LOS ICU) was not used due to lack of complete data and several confounding factors. No data were available to calculate the exact time from accident to transfusion. To indirectly control for substantial changes of this parameter we calculated the time from accident to arrival in OUHU for all trauma patients in each of the whole years of 2002, 2004 and 2007. We also calculated the frequency of patients arriving directly from the accident scene.

### Study design and data collection

The study including extraction and analysis of data was approved as a quality-assessing project. The following data were extracted from the hospital based trauma registry; Anatomic injury according to Injury Severity Score (ISS) and New Injury Severity Score (NISS) [13], both based on coding of anatomic injury according to The Abbreviated Injury Scale 1998, AIS 98 [14]. Physiological derangement on admission by Revised Trauma Score (RTS; the variables Glasgow Coma Scale score, respiratory rate and systolic blood pressure) [15]. Moreover, we extracted age, gender, type of injury, and outcome (30-day mortality) [16] for all trauma patients during the first 6 months of the years 2002, 2004, and 2007. Number of transfused units of erythrocytes, plasma, platelets, and haemoglobin values were registered for the first 10 days of hospital stay. When more than one haemoglobin value was recorded per day, the lowest one was used for this study. Transfusion algorithms for the respective periods were studied. The subpopulation of massively transfused patients (10 or more units of erythrocytes in 24 hours) was identified and analysed separately using Trauma Injury Severity Score (TRISS) methodology [17].

### Change in clinical practise in the study period

Transfusion practise in the year 2002 followed widely accepted principles [18]; blood loss up to one blood volume (equals 10 units of erythrocytes) was corrected with crystalloids, colloids and erythrocytes. In the interval 10 to 20 units of erythrocytes transfused, 1 unit of plasma was administered per 4 units of erythrocytes. This ratio was continued as long as massive transfusion protocol went on. Following transfusion of 15 units of erythrocytes, 1 unit of platelets was administered per 5 units of erythrocytes. The consultant anaesthesiologist in the trauma team used a variety of clinical criteria when initiating massive transfusion, but no formal protocol existed. In 2004, more focus was set on the need for immediate administration of erythrocytes in the emergency department in patients arriving in haemorrhagic shock, and to prevent hypothermia high capacity blood warmers (Level One^®^, Smiths Medical Inc.) were introduced. From 2006, damage control resuscitation (DCR) gained more awareness among surgeons and anaesthesiologists in the trauma team following publications from the United States [19-21]. Scandinavian guidelines in massive transfusion and achievements in massive transfusion presented from Denmark influenced our decisions [22,23]. Our totally revised massive transfusion protocol - following DCR principles - was implemented and practised from late 2006, including the delivery of "trauma packages" from the blood bank on request. Each package contains 5 units of erythrocytes, 5 units of plasma and 2 units of platelets.

### Statistical Analysis

Data were analyzed using a spreadsheet (Excel) and the statistical package EPI-INFO (CDC, WHO). The chi square test and Fisher's exact test were used for comparing frequencies. Mann-Whitney/Wilcoxon Two-Sample test was employed for other nonparametric data. Probability of Survival (Ps) was calculated using TRISS methodology - a logistic regression model based on the Major Trauma Outcome Study (MTOS), where the prediction variables are Revised Trauma Score (RTS), Injury Severity Score (ISS), age-index, and mechanism of injury (blunt/penetrating) [13-15]. W-statistic (expressing excess survivors per 100 patients treated at OUHU compared to TRISS model predictions) was calculated according to convention [17]. Updated coefficients from the US National Trauma Data Bank (NTDB) in 2005 were used [24].

## Results

### Population characteristics

The number of admitted trauma patients increased by 48% (149 patients) from 2002 to 2007 (Table 1). There was no significant change in ISS, NISS, RTS, age, gender, or mortality rate when comparing the whole trauma populations as well as the populations of transfused patients from the three periods. Damage control surgery, including emergency thoracotomy and/or laparatomy, was performed in 16 patients in the 2002 period, 15 patients in the 2004 period and 11 patients in the 2007 period. OUHU participated in a randomized study evaluating recombinant Coagulation Factor VIIa in uncontrolled through 2007, but very few patients were included. The product was not used therapeutically in the three periods studied.

**Table 1:** The trauma population at Oslo University Hospital Ullevål in the first six months of 2002, 2004 and 2007.

	First six months of 2002		First six months of 2004		First six months of 2007	
	All	Transfused day 1-10	All	Transfused day 1-10	All	Transfused day 1-10
**Number of trauma patients**						
Total (% transfused)	315	88 (28%)	341	96 (28%)	459	107 (23%)
						
**Age**						
Mean (SD)	33.8 (18.2)	37.8 (21.0)	33.7 (18.0)	39.9 (21.3)	34.5 (18.2)	39.9 (19.4)
						
**Gender (male)**						
N (%)	240 (76%)	68 (79%)	302 (70%)	76 (65%)	336 (74%)	76 (71%)
						
**Non-survival**						
N (%)	25 (8%)	15 (17%)	40 (9%)	27 (23%)	33 (7%)	21 (20%)
						
**ISS**						
Mean (SD)	14.9 (15.6)	29.9 (15.6)	15.2 (13.9)	31.5 (14.3)	14.7 (14.3)	30.7 (13.1)
						
**RTS**						
Mean (SD)	7.0 (1.5)	6.4 (1.8)	7.1 (1.4)	6.4 (1.8)	7.2 (1.3)	6.3 (1.8)

ISS = Injury Severity Score. Calculations based on coding according to AIS 98 (see reference [14] for details of ISS calculation). RTS = Revised Trauma Score (see reference [15] for details of calculation).

### Timing of transfusions

The logistics and procedures for preparation of blood products have been unchanged during the study period. Thus time from order to delivery of erythrocytes and platelets in the trauma room is likely to have been unchanged during the study period and is approximately 10 minutes including transport. The time from order to delivery of plasma is, following the same reasoning, unchanged at approximately 30 minutes. The longer time for this product is because prethawed plasma is not available. When a critically unstable patient is reported by the emergency medical service (EMS) erythrocytes stored in the trauma bay can be prepared ready for transfusion upon arrival of the patient. Trauma packages (including plasma) can also be requested before arrival of the patient to reduce delay in balanced transfusion. The latter is dependent on early report from the EMS.

### Time from accident to transfusion

The percentage of trauma patients arriving directly from the scene decreased from 88.9% in whole year of 2002, 85.3% in 2004 to 80.7% in 2007. Mean time from accident to arrival in the trauma room (for patients transported directly) was 1 hour 21 minutes in year 2002, 1 hour 42 minutes in 2004 and 1 hour 12 minutes in 2007.

In the groups of transfused patients the mean number of erythrocyte units given day 1-10 decreased from 9.4 in 2002 to 6.8 in 2007 (Table 2). This change was not statistically significant (p = 0.056). The changes in mean units of plasma and thrombocytes given day 1-10 were small. However, the ratio of total consumption of erythrocytes to total consumption of plasma decreased from 5.2 in 2002 to 3.5 in 2004 (p < 0.001) and further to 2.5 in 2007 (p = 0.02). The ratio of total consumption of erythrocytes to total consumption of thrombocytes decreased from 16.3 in 2002 to 9.4 in 2007 (p = 0.004), but not significantly from 10.8 in 2004 to 9.4 in 2007 (p = 0.44). A similar trend was seen in the subpopulation of massively transfused patients.

**Table 2:** Consumption of blood products day 1-10 after the trauma incident for patients admitted in the first six months of 2002, 2004 and 2007.

	First six months of 2002	First six months of 2004	First six months of 2007	Relevant p-values
**Number of patients transfused with erythrocytes day 1-10**				
N (% of whole trauma population)	88 (28%)	96 (28%)	107 (23%)	
				
**Consumption of erythrocytes**				
Total (median, mean units per. patient)	842 (5, 9.4)	834 (7.5, 8.7)	729 (5, 6.8)	2002 vs 2007: p = 0.056
**Number of patients given plasma**				
Total (percentage of transfused patients)	26 (30%)	37 (39%)	44 (41%)	2002 vs 2004: p = 0.38
				2004 vs 2007: p = 0.26
**Consumption of plasma**				
Total (median, mean units per. patient)	162 (4.5, 6.2)	270 (4, 7.3)	296 (4, 6.7)	
				
**Number of patients given trombocytes**				
Total (percentage of transfused patients)	15 (17%)	19 (22%)	22 (21%)	2002 vs 2004: p = 0.71
**Consumption of trombocytes**				
Total (median, mean units per. patient)	51.5 (3, 3.4)	77 (2, 3.6)	77.5 (2, 3.5)	
				
**Total units of erythrocytes consumed by massively transfused patients day 1-2**				
Total (% of total consumption)	283 (33.6%)	356 (42.7%)	171 (23,5%)	
				
**No of erythrocyte unites per massively transfused patient**				
	21.8	18.7	19.0	

The table lists total consumption and relevant data for the subgroup of patients receiving the different types of blood products. For comparing consumption of erythrocytes, plasma and trombocytes Mann-Whitney test was employed. Both median and mean values are reported.

Massive transfusion patients contributed greatly to the consumption of blood products, especially in 2004, when more than 40% of the consumption of erythrocytes was due to treatment of the 20 patients transfused with 10 or more units during the first 24 hours. Significantly fewer patients were massively transfused during the first six months of 2007 compared to 2004 (p = 0.008). When comparing 2007 to 2002 there was a non-significant decrease (p = 0.11). Massively transfused patients are characterised by a high ISS, low RTS and a high mortality rate (Table 3).

**Table 3:** Characteristics and survival rates for the population of patients that were massively transfused day 1-2 after admittance.

	First six months of 2002	First six months of 2004	First six months of 2007
**Number of patients**	13	19	9
Proportion of all transfused patients (%)	13/62 (21%) **	19/74 (26%)**/*	9/94 (10%)*
			
**Age**			
Mean (SD)	33.5 (21.7)	34.8 (16.5)	45.2 (14.0)
			
**Gender (male)**			
N (%)	10 (76.9%)	15 (75.0%)	14 (73.7%)
			
**Non-survival**			
N (%)	4 (30.8%)	8 (40.0%)	3 (33.3%)
			
**ISS**			
Mean (SD)	38.5 (18.2)	42.0 (16.6)	41.1 (11.2)
			
**RTS**			
Mean (SD)	5.5 (2.0)	5.2 (2.2)	5.9 (1.8)

		* p = 0.002	** p = 0.10

Massive transfusion is defined as transfusion of more than 10 units of erythrocytes in 24 hours. ISS = Injury Severity Score. RTS = Revised Trauma Score. Fisher's exact test was used for comparing frequencies.

Median and mean New Injury Severity Score (NISS) for both transfused patients and massively transfused did not increase significantly (Table 4).

**Table 4:** W-statistic based on TRISS comparing all trauma patients, transfused patients and massively transfused patients day 1-2.

	All trauma patients			Transfused trauma patients			Massively transfused trauma patients		
First six months of	2002	2004	2007	2002	2004	2007	2002	2004	2007
**Number**	315	341	459	62	74	94	13	19	9
									
**Non-survival**N (%)	24(7,6)	34(10,0)	34(7,4)	12(19,4)	22(29,7)	19(20,2)	4(30,8)	8(42,1)	3(33,3)
									
**W-statistic**	1,98	0,34	0,66	3,99	3,9	5,18	15,3	8,89	9,76
(NTDB 05)									
									
**95% C.I**	(-0,15 -4,12)	(-1,66 -2,34)	(-1,03 -2,35)	(-3,21 -11,19)	(-3,08 -10,88)	(-0,92 -11,28)	(-2,40 -33,0)	(-6,47 -24,26)	(-13,87 -33,4)
									
**ISS**median	10	10	10	27	29,5	29	38	43	38
									
**ISS**mean	14,9	15,2	14,7	29,9	31,5	30,7	38,5	42	41,1
									
**NISS**median	11	13	12	34	38	41	43	50	57
									
**NISS**mean	19,3	20	19	39,6	40,5	41,5	42,8 */**	48,7 *	52,6 **

				* p = 0.36			** p = 0.24		

ISS = Injury Severity Score. RTS = Revised Trauma Score.

### Haemoglobin values recorded during the first 10 days after admittance

**Figure 1:**
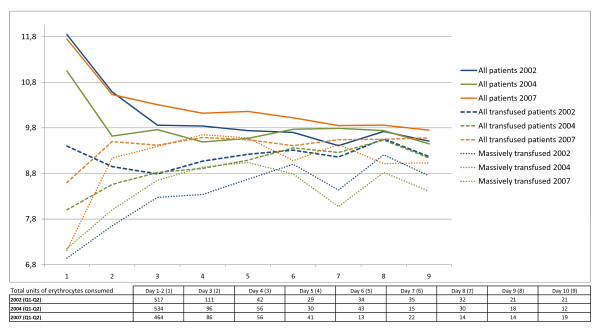
**Mean haemoglobin values for the groups of all trauma patients, transfused trauma patients and massively transfused trauma patients day 1-10 after admittance**. Total units of erythrocytes administered each day to the transfused patients are listed in the separate table below the figure.

Mean haemoglobin values for each day in the whole population of trauma patients were not significantly different at any day during the 10-day period after admittance when comparing the patient groups from 2002, 2004 and 2007. The mean value seemed to stabilize around 9-9.5 g/dL (Figure 1).

In the group of transfused patients the mean haemoglobin value day 1-2 after the accident in 2002 (9.52 g/dL) was significantly higher than in 2004 (8.04 g/dL, p < 0,001) and in 2007 (8.55 g/dL, p = 0,005) (Figure 1). The next day, however, a marked reduction in mean haemoglobin in 2002 was noticed while mean haemoglobin in 2007 increased. Thus, on day 3 mean haemoglobin in 2007 (9.45 g/dL) was significantly higher than in 2002 (8.9 g/dL, p = 0,0013) and in 2004 (8.7 g/dL, p < 0.001). Mean haemoglobin value in 2007 remained significantly higher than in 2002 and 2004 until day 6 after trauma incident. No significant difference was found when comparing the values on day 7-9, but on day 10 mean haemoglobin in 2007 (9.53 g/dL) was significantly higher than in 2004 (9.06 g/dL, p = 0.022) and in 2002 (9.12 g/dL, p = 0.023).

The proportion of patients transfused with one or two units of erythrocytes showed little change during the 5-year period (26.1%, 29.2% and 29.0%).

### 30-day mortality

Overall 30-day mortality was slightly better in 2007 than in 2002 and 2004, but the change did not reach statistical significance. No significant changes were found when comparing groups of patients minimally, moderately or massively transfused. Using TRISS-methodology no significant change in W-statistic (excess survivors per 100 patients) was found in any group (Table 4).

## Discussion

We have shown that significant changes of transfusion practice has occurred during the past decade, probably as a result of increased focus on the need for early haemostasis and more precise criteria for initiation of massive transfusion. However, despite a lower consumption of erythrocytes in 2007 than in 2002 and 2004, the mean haemoglobin level of transfused patients was higher on day 10 in 2007.

### Trauma activity

The number of trauma patient admissions increased by 48% from 2002 to 2007. Despite this increase, no significant change in core variables like ISS, RTS, age and survival rate was found. The proportion of patients with severe injury was also unchanged (40% in the whole period). For transfused and massively transfused patients, the apparent increase of NISS failed to reach statistical significance. Thus the patients receiving erythrocytes were not more seriously injured in 2004 and 2007 (Table 4).

During the five-year period major changes in the organisation of the hospitals in central parts of Norway occurred. Key data from the national statistical service does not indicate a marked increase of number of accidents from 2002 to 2007 - in fact the number of severely injured patients in road traffic accidents decreased from 1329 to 828 in the 10-year period from 1998 to 2008. The number of patients with moderate injury also decreased from 10800 to 7300 according to the national statistical service. An extra physician-manned (anaesthesiologist) emergency medical helicopter was assigned to the region from the summer of 2002. This significant increase in helicopter transport capacity may have facilitated transport of more trauma victims to OUHU. The increased proportion of patients arriving from other hospital may be a natural finding given the increase of the total number of trauma patients. We think this reflects that more patients that otherwise would have been treated in smaller hospitals are transferred to the trauma hospital. We believe that OUHU has become more of a regional and national trauma centre during the study.

In Scandinavia efforts have been made to unite on guidelines for massive transfusion [22]. Norway generally has the lowest consumption of blood products *per capita *of the Nordic countries [25] - a fact that is interesting enough to merit further investigation, also on the use of transfusion in trauma care.

### Time from accident to transfusion

Because of lack of precise data our calculation based on the whole trauma population in the years of 2002, 2004 and 2007 must be interpreted with care. Mean time seems to increase in 2004 and decrease in 2007

### Procurement and consumption of blood products

The blood products used at OUHU are units of erythrocytes (mean volume of 1 unit of erythrocytes is 245 ml, and hct is on average = 55), plasma (all hospitals in Norway use the product Octaplas^® ^instead of single unit fresh frozen plasma. Mean volume of 1 unit is 200 ml). Our platelet units (volume ca. 350 ml) contain platelets from 4 donors of similar blood ABO and Rh(D) blood groups mixed together. All units of erythrocytes and platelets were leukocyte filtrated before storage. To our knowledge, Octaplas^® ^has never been reported to induce transfusion related acute lung injury (TRALI). This probably results from dilution and neutralisation of TRALI-inducing antibodies in the production process. Octalpas^® ^is produced by pooling of 3000 units of FFP for preparation of each Octaplas batch.

### Consumption of blood products

The decrease in the ratios of erythrocytes to plasma as well as to thrombocytes is in accordance with modern guidelines for transfusion in trauma patients [22]. No formal change in local guidelines occurred from 2002 to 2004, and our results may therefore reflect that clinicians change their practice according to evidence before formal guidelines are revised.

Massively transfused patients contributed largely to the consumption of erythrocytes and plasma in all three periods studied, but the number of massively transfused patients decreased significantly from 2004 to 2007. This may explain the small change in consumption of thrombocytes. The reduced use of massive transfusion may reflect improvements in trauma care like earlier use of DCR principles (permissive hypotension prior to definitive surgery, damage control surgery including angiographic embolization techniques, avoidance of hypothermia), and increased focus on acute traumatic coagulopathy and haemostatic resuscitation [26-28].

Almost 8% of patients (2007) received one or two units of erythrocytes. Transfusion of such small volumes is controversial because the increase in haemoglobin value is small, while the hazards of transfusion persist [29-32]. Some of these transfusion episodes may have occurred because the clinical diagnosis of hypovolaemic shock in the trauma room is uncertain and that some transfusions are aborted when the first blood samples are analyzed and early stabilisation of the patient is obtained.

### Haemoglobin trends

The mean lowest haemoglobin value was significantly higher on day two in 2002 compared to day two in 2007. This may reflect differences in the way the first blood sample was provided or differences in the amount of fluids given, but also that the practice of erythrocyte transfusion has become more restrictive. The change in mean number of units of erythrocytes given to the transfused patients in 2002 and 2007 is marked and supports the latter assumption, although not statistically significant (p = 0,056). The somewhat reduced percentage of patients who were transfused, may further support this interpretation. Unfortunately, we were not able to obtain sufficient data about the infusion of fluids in the pre-hospital phase and in the trauma room. The haemoglobin values will be influenced by changes in amounts of fluids given.

In accordance with the observations of Vincent et al. our results illustrate that the mean haemoglobin values tend to stabilize 3-4 days after admittance at values around 9-9.5 g/dL [33]. It is tempting to propose that this reflects an adaptation of the production of erythrocytes to the situation of the intensive care unit patient, reducing blood viscosity to facilitate microcirculation [34].

### Why does the clinical practice change?

There are probably several reasons for the reduced use of erythrocytes. A more restrictive use of infusions in the pre-hospital phase during recent years may present the team with trauma patients that have a slightly higher primary haemoglobin values. It is also possible that the increased use of plasma and platelets in the early phase of treatment improves coagulation and thus reduces the total blood loss. A more restrictive use of fluids in the hospital may reduce the total blood loss and thus decrease the need for erythrocytes. Unfortunately, we do not have precise data about the amount of fluids given in any phase of treatment. In addition, increased use of arterial blood samples (blood gas analyzers have been installed in the ED and operation unit during the study period) could give the clinicians the possibility to reduce the number of transfused units when adequate haemoglobin level is noted.

### Mortality

Several retrospective reports exist which indicate that aggressive use of prohaemostatic blood products reduce mortality in bleeding trauma patients [35,36]. Others have failed to find such a correlation [7,9]. In our study mortality was low at the outset, and only relatively small changes might be expected to occur. Also, and especially for massively transfused patients, the number of patients included may be too low to show any change. Prospective studies, preferably randomized clinical trials with large enough patient groups and strict control with influencing factors, are needed to reach a conclusion on the effect of pro-haemostatic blood products in trauma patients [8].

The increased use of DCS and radiological interventions could be thought to increase survival rates in our material, but the number of patients receiving this treatment is low and a possible effect on mortality would probably not be reflected because we compared short periods of six months. In another study from our hospital a significant increase in survival rates for the whole trauma population in has been reported [37].

Our results support what Dutton and co-workers point out in a large study of trauma mortality patterns in a ten year material [38]. Improved survival in prospective randomized trials is difficult to find because of the low mortality in modern trauma centres and the small number of patients in whom outcome can be influenced. New knowledge on post-injury haemostasis and implementation of goal-directed approach to post-injury coagulopathy may provide more answers in the future [39].

### Limitations of the study

This study has limitations due to patient number and lack of some key data that would be valuable to our analysis. Even if there were major positive changes in transfusion therapy and total quality of trauma care, the likelihood of this being reflected as changed mortality outcome in a survey of this size is small. One important reason for this is that only a small fraction of the transfused patients are massively transfused and in need of a modern balanced ratio of blood components to increase survival. We do, however, believe it is methodically correct to analyze for such changes despite these assumptions.

Exact time from accident to arrival in the trauma room would be of importance, because the timing of transfusion is of importance. Unfortunately the time can only be estimated due to lack of complete data in our trauma registry. Exact data regarding pre-hospital and in-hospital volumes of infused fluids would also be of great interest and valuable when interpreting the changes in haemoglobin and transfusion found in our data.

### Do improvements in other parts of trauma care affect our results?

In 2004 highly efficient blood and fluid warmers were introduced at OUHU, thus reducing the hypothermic effect of massive transfusions and infusions, and improving the conditions for efficient haemostasis. In the same period a 24/7/365 service of haemostatic angiographic embolization became available. This service may have reduced the number of massively bleeding patients. The increased focus on early external fixation of pelvic fractures and the use of a high-quality and faster CT-facility may also be influential. In addition the constant training and increased use of video-feedback in the trauma team may improve quality of care.

### Resource considerations

Consumption of blood products is increasing in many countries, Norway included [24,40]. It is interesting, therefore, to note that a reduced consumption of erythrocytes in the treatment of trauma had no negative effect on 30-day mortality. This should encourage attempts at reducing erythrocyte consumption also for other patient groups in order to avoid shortage of blood supply.

## Conclusions

Significant changes of transfusion practice occurred during the five year period studied, possibly as result of increased multimodal focus on haemostasis and as a result of new transfusion algorithms reflecting such a focus. Despite a lower consumption of erythrocytes in 2007 than in 2002 and 2004, which was probably reflected in a lower mean haemoglobin value on day two, the mean haemoglobin level of transfused patients was higher on day 10 in 2007. This may reflect a more restrictive practice of fluid resuscitation or improvements in other parts of trauma care. The reduced consumption of erythrocytes is valuable *per se*, since shortage of erythrocyte supply is threatening due to an ageing population in general and difficulties of recruiting and retaining blood donors [36].

High plasma - and platelets to erythrocyte ratios have been reported to improve survival in patients with massive bleeding [41]. Like some other studies our results fail to support this, but the effect of this therapeutic approach must be subject to future studies of larger patient groups with strict control of all influencing factors before final conclusions are drawn.

## Competing interests

None of the authors have any conflict of interest with regard to the material discussed in this manuscript.

## Authors' contributions

HEH, JPL and NOS made the first analysis on data from 2002. HEH designed the study. NOS generated the data from the trauma registry. BG generated the data from the Blood Bank. ARN merged the data for all three periods and performed statistical analysis. NOS performed the TRISS-analysis. ARN was responsible for making figures and tables. All authors participated in the writing process. All read and approved the final manuscript.
